# Treatment Adherence With an Oral 9-Month Regimen for Rifampicin-Resistant Tuberculosis in South Africa

**DOI:** 10.1093/cid/ciag069

**Published:** 2026-02-09

**Authors:** Johanna Kuhlin, Jacob A M Stadler, Daniel J Grint, Richard Court, Graeme Meintjes, Nomfuneko Mtwa, Gary Maartens, Sean Wasserman

**Affiliations:** Department of Medicine Solna, Karolinska Institutet, Stockholm, Sweden; Department of Infectious Diseases, Karolinska University Hospital, Stockholm, Sweden; Wellcome Discovery Research Platforms in Infection, Centre for Infectious Diseases Research in Africa, Institute of Infectious Disease and Molecular Medicine, University of Cape Town, Cape Town, South Africa; Wellcome Discovery Research Platforms in Infection, Centre for Infectious Diseases Research in Africa, Institute of Infectious Disease and Molecular Medicine, University of Cape Town, Cape Town, South Africa; Department of Medicine, University of Cape Town, Cape Town, South Africa; Department of Infectious Disease Epidemiology, London School of Hygiene and Tropical Medicine, London, United Kingdom; Division of Clinical Pharmacology, Department of Medicine, University of Cape Town, Cape Town, South Africa; Wellcome Discovery Research Platforms in Infection, Centre for Infectious Diseases Research in Africa, Institute of Infectious Disease and Molecular Medicine, University of Cape Town, Cape Town, South Africa; Department of Medicine, University of Cape Town, Cape Town, South Africa; Blizard Institute, Queen Mary University of London, London, United Kingdom; Department of Health, Eastern Cape Province, East London, South Africa; Wellcome Discovery Research Platforms in Infection, Centre for Infectious Diseases Research in Africa, Institute of Infectious Disease and Molecular Medicine, University of Cape Town, Cape Town, South Africa; Division of Clinical Pharmacology, Department of Medicine, University of Cape Town, Cape Town, South Africa; Wellcome Discovery Research Platforms in Infection, Centre for Infectious Diseases Research in Africa, Institute of Infectious Disease and Molecular Medicine, University of Cape Town, Cape Town, South Africa; Institute for Infection and Immunity, City St George's, University of London, London, United Kingdom

**Keywords:** adherence trajectories, 9- to 12-month regimen, risk factors, digital pillbox, latent class group-based trajectory modeling

## Abstract

**Background:**

Adherence to anti-tuberculosis (TB) therapy is an important determinant of treatment outcome in rifampicin-resistant tuberculosis (RR-TB). Understanding adherence to contemporary treatment regimens in routine care is needed to support implementation in TB programs. We aimed to characterize temporal adherence patterns among people receiving oral treatment for RR-TB.

**Methods:**

We conducted a prospective observational cohort study at a referral TB hospital in South Africa. People aged ≥15 years with pulmonary RR-TB starting an oral 9- to 12-month regimen were included. Treatment adherence was measured using a digital pillbox during ambulatory care and with directly observed therapy during hospital care. The primary outcome was proportion of adherence days through 9 months. Latent class group-based trajectory modeling was used to identify temporal adherence patterns.

**Results:**

Of 248 participants, 209 (84.3%) had assessable adherence data from the digital pillbox or directly observed therapy. Overall median adherence was 82% (interquartile range [IQR], 63%–98%) with combined measures, and 72% (IQR, 51%–92%) with digital pillbox only. Four distinct adherence patterns were identified. Adherence was 93%–100% in the first month. Two groups, representing 136 (65.1%) individuals, had small reductions in adherence over time, separated by higher and lower early adherence. In the other 2 groups, there was a 50% reduction in adherence by month 3 (48/209 [23.0%]) and month 6 (25/209 [12.0%]), respectively. Lower adherence over time was associated with having exclusive ambulatory care, treatment with the shorter regimen only, and age <40 years.

**Conclusions:**

Treatment adherence declined over time in distinct temporal patterns. Group characteristics could identify individuals who may benefit from enhanced treatment support.

## Teaser

Electronic pillboxes were used to measure treatment adherence for rifampicin-resistant tuberculosis. Adherence declined over time in 4 distinct temporal patterns. Lower adherence was associated with having exclusive ambulatory care, treatment with the shorter regimen only, and age <40 years.

Treatment adherence is a major determinant of tuberculosis (TB) treatment outcomes [1] and is particularly important in rifampicin-resistant TB (RR-TB) [2], for which anti-TB drugs are less effective. In 2024, RR-TB affected an estimated 390 000 people globally, with only 71% achieving treatment success [3]. Decreased adherence to treatment for RR-TB, mainly using injectable-based regimens, has been associated with lack of sputum culture conversion (SCC) [4, 5] and increased risk of poor outcomes [4, 6], including acquired drug resistance [6] and death [5].

Overall adherence to these longer injectable-based regimens in programmatic settings has been reported between 80% and 87% over 6 months [7]. However, there is large heterogeneity in how adherence is measured and reported. Previous approaches have included the proportion with at least 85%–90% intake [5, 8] or missing at least 2 days [6]. Such cutoffs inadequately capture detailed temporal adherence patterns, which are important for understanding drivers and defining potential interventions for poor adherence [6], and may be more predictive of treatment outcomes [9].

Previous prospective studies analyzing programmatic treatment adherence in RR-TB have been limited by unreliable ascertainment (such as use of self-reporting [10] or pill counts [9]), short follow-up duration (usually 6 months only [5, 7, 11]), use of cross-sectional designs [10, 12], or analysis of outdated individualized treatment regimens [7, 11], which are less relevant with several oral shorter regimens now available [2, 13]. Retrospective studies usually ascertain adherence through directly observed treatment (DOT) [4, 6, 8, 14], introducing bias as it also provides adherence support, and may limit generalizability in resource-constrained settings. Few studies have used objective adherence measurements beyond DOT in RR-TB, such as use of digital pillboxes [5, 7], which demonstrate high reliability in RR-TB [15].

There is limited information on adherence patterns over the full course of oral RR-TB treatment in programmatic settings. Understanding adherence patterns over time using a reliable measure, and delineating associated risk factors, could guide healthcare workers and programs in directing enhanced adherence support to those most in need. We aimed to describe adherence trajectories over the full treatment period to identify individuals at risk of poor adherence with an oral shorter bedaquiline-based regimen for RR-TB.

## METHODS

### Design

We conducted a prospective observational cohort study (SHort-course oral regImen For ressitant Tb, SHIFT-TB) among people starting treatment for RR-TB at a referral TB hospital in Eastern Cape Province, South Africa [16]. Consenting individuals 15 years and older with pulmonary RR-TB starting a standardized oral 9- to 12-month regimen were included and followed up at 7 study visits over 12 months. This regimen was standard of care in South Africa during the study (January 2021–August 2022) and comprised 7 oral drugs; bedaquiline, levofloxacin, high-dose isoniazid, clofazimine, pyrazinamide, and ethambutol, with linezolid provided for the first 2 months [17]. Some participants were later switched to an individualized longer regimen according to national RR-TB guidelines [16, 17]. Treatment was administered by staff in routine care.

Adherence measurements included DOT during hospitalization and a digital pillbox (EvriMed 500, Wisepill, Somerset West, South Africa) during outpatient care. All anti-TB drugs were packed in the digital pillbox by study personnel. The pillbox could hold approximately 1 month’s supply of drugs and was refilled during study visits; if study visits were further apart than 1 month, participants were counseled to refill the pillbox themselves. Participants were trained on use of the digital pillbox, including instruction to open only for drug intake. Pillbox opening information required data download directly from the pillbox, which had no activated reminders or alarms. Routine care adherence support was provided to all participants, including counseling and referrals for substance use or mental health concerns [17]. Transport to clinic visits was not reliably provided. If poor adherence was indicated from the digital pillbox data, participants were referred to a hospital-based social worker for enhanced support (which included counseling and adherence group sessions), but the study itself did not provide additional adherence support.

Written informed consent was obtained before study procedures. Ethical approval was granted by the University of Cape Town Human Research Ethics Committee (REF. 690/2019) and the Eastern Cape Department of Health (EC_201911_017).

### Definitions

An adherence day was defined from 4:00 Am until 3:59 Am the following day, assuming that pillbox openings (a proxy for drug intake) after midnight until 3:59 Am represented a previous day’s intake. Drug intake referred to any intake with DOT or recorded pillbox opening when drugs were prescribed. The digital pillbox recorded daily electronic signals to confirm function. An assessable adherence day was defined as days with this electronic signal or provision of DOT when drugs were prescribed. Treatment interruption was defined as no drug intake for at least 1 day during assessable adherence days. Prescriber-initiated drug stoppages (eg, for adverse reactions) were excluded.

Adherence was measured until the first occurrence of (1) treatment outcome using the World Health Organization (WHO) 2021 definition (Supplementary Methods) [2, 16]; (2) transfer of care to another facility; or (3) completion of 9 months of treatment. Participants with regimen changes due to acquired resistance or treatment-related factors were assigned an unfavorable treatment outcome and adherence measurements were discontinued.

### Statistical Analysis

Assessable adherence days were reported as the proportion of assessable adherence days out of all potential adherence days. Adherence was calculated as the proportion of drug intake days out of assessable adherence days, monthly (30-day periods) and overall. We performed latent class group-based trajectory modeling on monthly adherence to identify distinct groups (see details in the Supplementary Methods). Patterns of treatment interruptions and gaps between interruptions were summarized overall and by trajectory group.

Multinomial logistic regression was performed to explore predictors of trajectory group membership. Potential confounders in regression modeling were selected from previous TB studies (age, sex, receiving social grants, higher education, previous TB [5, 8, 18–20]) and possible sociodemographic and clinical factors (working/studying, being single, receiving shorter regimen only, ambulatory care only, human immunodeficiency virus [HIV] test positivity, disease severity factors [baseline albumin, hemoglobin, and positive microscopy], and alcohol use). Forward selection added predictors with at least *P* < .1 (in multinomial regression *P* < .1 to at least 1 group), retaining only associated factors with *P* < .05 in the multivariable model. Marginal probabilities for group membership were estimated from the multinomial logistic regression model using absolute probability change between predictor combinations.

Logistic regression and Cox proportional hazard models were used to analyze relationships between overall adherence and trajectory group membership (exposures) with loss to follow-up (LTFU), treatment failure, death, and time to sustained SCC (outcomes) (see Supplementary Methods for definitions). Data were censored at the end of follow-up.

Missing adherence data from technical issues (eg, battery, connectivity) and early treatment cessation (due to death or LTFU) was assumed missing at random (eg, was not associated with adherence in itself). A complete case analysis was used for predictors with ≤10% missing data, otherwise “unknown” categories were included. Sensitivity analyses were performed with only digital pillbox data (DOT excluded) and only participants with ≥75% assessable adherence.

The sample size for this adherence analysis was determined by the parent cohort study, which was powered for detecting treatment outcomes.

Analysis was done in Stata version 16.1 software (StataCorp, College Station, TX, USA).

## RESULTS

Of 248 participants in the cohort, 209 (84.3%) provided 37 953 assessable adherence days from the digital pillbox or DOT. Thirty-nine of the 248 participants (15.7%) had no assessable digital pillbox data (Supplementary Figure 1, Table 1); reasons included uncontactable participants (n = 21), lost pillbox (n = 6), forgotten pillbox (n = 9), and other reasons (n = 3).

**Table 1.** Participant Characteristics and Treatment Outcomes

Characteristic	All (N = 248)	Assessable Data From the Digital Pillbox or DOT Only (n = 209)	No Assessable Data From the Digital Pillbox (n = 39)
Age, y, median (IQR; range)	38 (31–45; 17–77)	38 (32–46; 17–66)	34 (30–40; 19–77)
Age <40 y	137 (55.2)	108 (51.7)	29 (74.4)
Female sex	102 (41.1)	81 (38.8)	21 (53.8)
HIV test positive	173 (69.8)	140 (67.0)	33 (84.6)
Positive microscopy	137 (55.2), n = 245	117 (56.0), n = 207	20 (51.3), n = 38
Positive sputum culture	200 (80.6), n = 244	168 (80.4), n = 206	32 (82.1), n = 38
Baseline hemoglobin, g/dL, mean (95% CI)	10.5 (10.2–10.8), n = 245	10.8 (10.5–11.1), n = 207	9.0 (8.4–9.6), n = 38
Baseline albumin, g/L, mean (95% CI)	29 (28–30), n = 240	30 (29–31), n = 201	26 (24–28), n = 39
Previous TB disease	117 (47.2)	99 (47.4)	18 (46.2)
Relationship status: single	213 (85.9)	176 (84.2)	37 (94.9)
Working/studying	87 (35.1)	75 (35.9)	12 (30.8)
Education level			
No education	3 (1.2)	2 (1.0)	1 (2.6)
Primary school	56 (22.6)	49 (23.4)	7 (17.9)
High school	170 (68.5)	139 (66.5)	31 (79.5)
University	19 (7.7)	19 (9.1)	0 (0.0)
Monthly household income (USD)^a^			
<55	52 (21.0)	43 (20.6)	9 (23.1)
55to <275	138 (55.6)	116 (55.5)	22 (56.4)
275 to <550	30 (12.1)	26 (12.4)	4 (10.3)
550 to <825	17 (6.9)	15 (7.2)	2 (5.1)
≥825	11 (4.4)	9 (4.3)	2 (5.1)
Received a social grant	87 (35.1)	75 (35.9)	12 (30.8)
CAGE score^b^ ≥2			
No	75 (30.2)	66 (31.6)	9 (23.1)
Yes	128 (51.6)	107 (51.2)	21 (53.8)
Missing	45 (18.1)	36 (17.2)	9 (23.1)
Ambulatory care only	86 (34.7)	81 (38.8)	5 (12.8)
Shorter regimen only	179 (72.2)	149 (71.3)	30 (76.9)
Treatment length total regimen, d, median (IQR)	287 (133–341)	292 (182–353)	126 (79–257)
Sustained SCC	186 (93.0)	156 (92.9)	30 (93.8)
Time to sustained SCC, d, median (IQR), n = 156	28 (15–42), n = 186	29 (16–42)	23 (14–35), n = 30
Treatment outcome			
Treatment success	126 (50.8)	118 (56.5)	8 (20.5)
Treatment failed	16 (6.5)	14 (6.7)	2 (5.1)
Lost to follow-up	74 (29.8)	50 (23.9)	24 (61.5)
Died	32 (12.9)	27 (12.9)	5 (12.8)

Data are presented as No. (%) unless otherwise indicated.

Abbreviations: CI, confidence interval; DOT, directly observed therapy; HIV, human immunodeficiency virus; IQR, interquartile range; SCC, sputum culture conversion; TB, tuberculosis; USD, United States dollars.

^a^One South African rand is 0.055 USD.

^b^CAGE is a 4-question screening tool for alcohol assessment.

Among the 209 participants with assessable adherence data, median age was 38 (interquartile range [IQR], 32–46) years, 38.8% (81/209) were female, and 67.0% (140/209) were HIV positive. Most participants were unemployed and unmarried; 76.1% (159/209) had a monthly household income of less than US$275. During treatment, 38.8% (81/209) received ambulatory care only and 71.3% (149/209) were treated with the shorter regimen only (ie, not switched to individualized longer regimens) (Table 1). At end of treatment, 56.5% (118/209) had a successful treatment outcome and 23.9% (50/209) were lost to follow-up. Differences among the 39 participants with unassessable compared with assessable pillbox data included younger age, more females and single relationship status, higher HIV positivity, and fewer receiving ambulatory care only.

**Figure 1.**
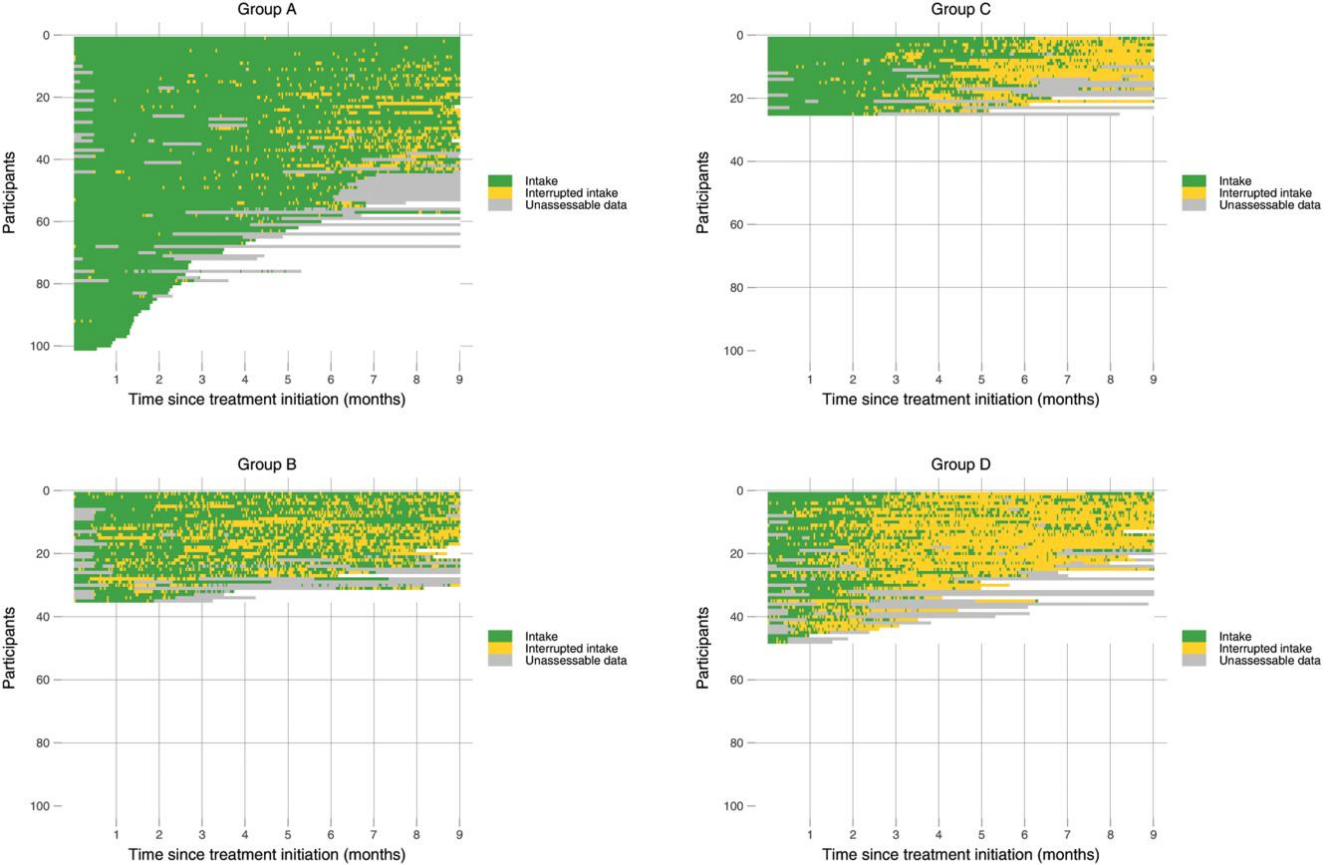
Daily drug intake, treatment interruption, and unassessable adherence data by adherence trajectory group. Follow-up duration was until the first occurrence of either (1) treatment outcome according to the World Health Organization; (2) transfer to another facility; or (3) completion of 9 months of treatment. Data are sorted by intake, interrupted intake, and unassessable adherence data.

**Figure 2.**
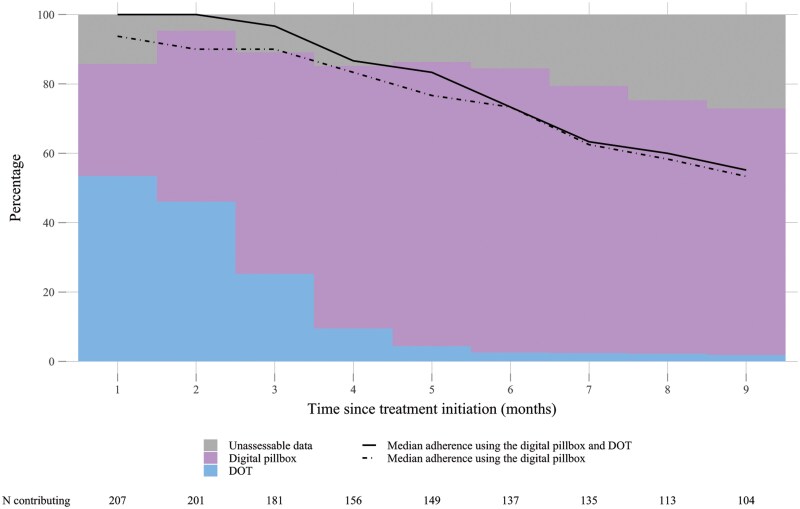
Observed monthly adherence. Adherence is measured until the first occurrence of either (1) treatment outcome according to the World Health Organization; (2) transfer to another facility; or (3) completion of 9 months of treatment. Abbreviation: DOT, directly observed treatment (given during hospital treatment).

Daily assessable adherence, drug intake, and treatment interruptions are shown in Figure 1. Assessable adherence days were available for 95% (IQR, 76%–100%) of follow-up time using digital pillbox and DOT combined, and for 92% (IQR, 67%–100%) using digital pillbox only (Supplementary Table 1): 103 (49.3%) with both DOT and pillbox data, 24 (12.0%) DOT only, and 81 (38.8%) pillbox only. Overall median adherence was 82% (IQR, 63%–98%) combining DOT and pillbox data, and 72% (IQR, 51%–92%) with pillbox data only (Figure 2, Supplementary Table 2).

**Figure 3.**
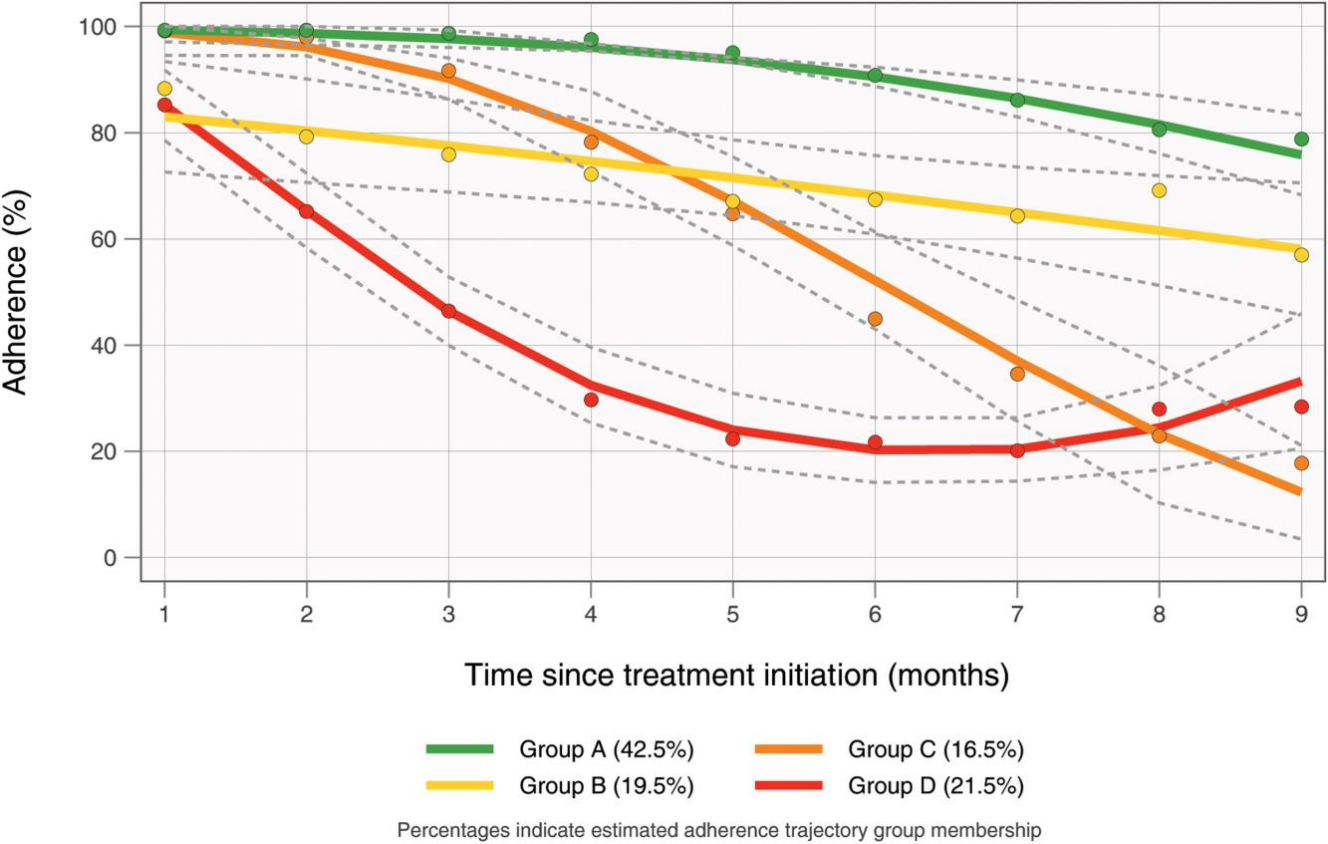
Adherence trajectories over 9 months. Solid lines are estimated adherence trajectories. Dotted lines indicate 95% confidence intervals. Dots represent observed group means at different months.

There were 4 distinct adherence patterns and groups (Figure 3, Supplementary Figure 2, Supplementary Tables 3 and 4). In the first month, observed adherence was 93%–100% in all groups (Table 2). Adherence decreased slightly over time in groups A and B, comprising 101 of 209 (48.3%) and 35 of 209 (16.7%) participants, respectively. Group B had lower early adherence but declined in parallel with group A. Group C, with 25 of 209 (12.0%) participants, had delayed, but substantial, decreases in adherence; group D, with 48 of 209 (23.0%) participants, showed immediate, large decreases. By month 9, median observed adherence was 10% in group C and 18% in group D (Table 2). Sensitivity analyses using digital pillbox data only or participants with ≥75% assessable adherence days showed similar 4-group patterns (Supplementary Figures 3 and 4).

**Table 2.** Monthly Observed Adherence by Adherence Trajectory Group Using the Digital Pillbox and Directly Observed Treatment

Adherence	Group A (n = 101), Median % (IQR)	Group B (n = 35), Median % (IQR)	Group C (n = 25), Median % (IQR)	Group D (n = 48), Median % (IQR)
Adherence overall	98 (92–100), n = 101	70 (62–81), n = 35	70 (63–74), n = 25	45 (34–58), n = 48
Adherence monthly				
Month 1	100 (100–100), n = 101	94 (83–81), n = 35	100 (100–74), n = 25	93 (77–58), n = 46
Month 2	100 (100–100), n = 97	87 (67–81), n = 35	100 (100–74), n = 25	65 (50–58), n = 44
Month 3	100 (100–100), n = 82	77 (60–81), n = 33	100 (90–74), n = 25	43 (30–58), n = 41
Month 4	100 (97–100), n = 68	70 (60–81), n = 30	80 (67–74), n = 23	30 (13–58), n = 35
Month 5	97 (93–100), n = 64	68 (48–81), n = 28	73 (37–74), n = 23	18 (3–58), n = 34
Month 6	97 (87–100), n = 58	68 (55–81), n = 28	37 (13–74), n = 22	17 (3–58), n = 29
Month 7	93 (80–100), n = 58	66 (47–81), n = 30	32 (8–74), n = 20	13 (7–58), n = 27
Month 8	87 (63–100), n = 46	71 (49–81), n = 28	12 (3–74), n = 16	23 (0–58), n = 23
Month 9	85 (62–100), n = 44	58 (38–81), n = 24	10 (0–74), n = 15	18 (13–58), n = 22

Adherence is calculated over all assessable adherence days (eg, days with an electronic signals to confirm function from the digital pillbox or directly observed treatment days). Observed adherence is reported.

Abbreviation: IQR, interquartile range.

**Figure 4.**
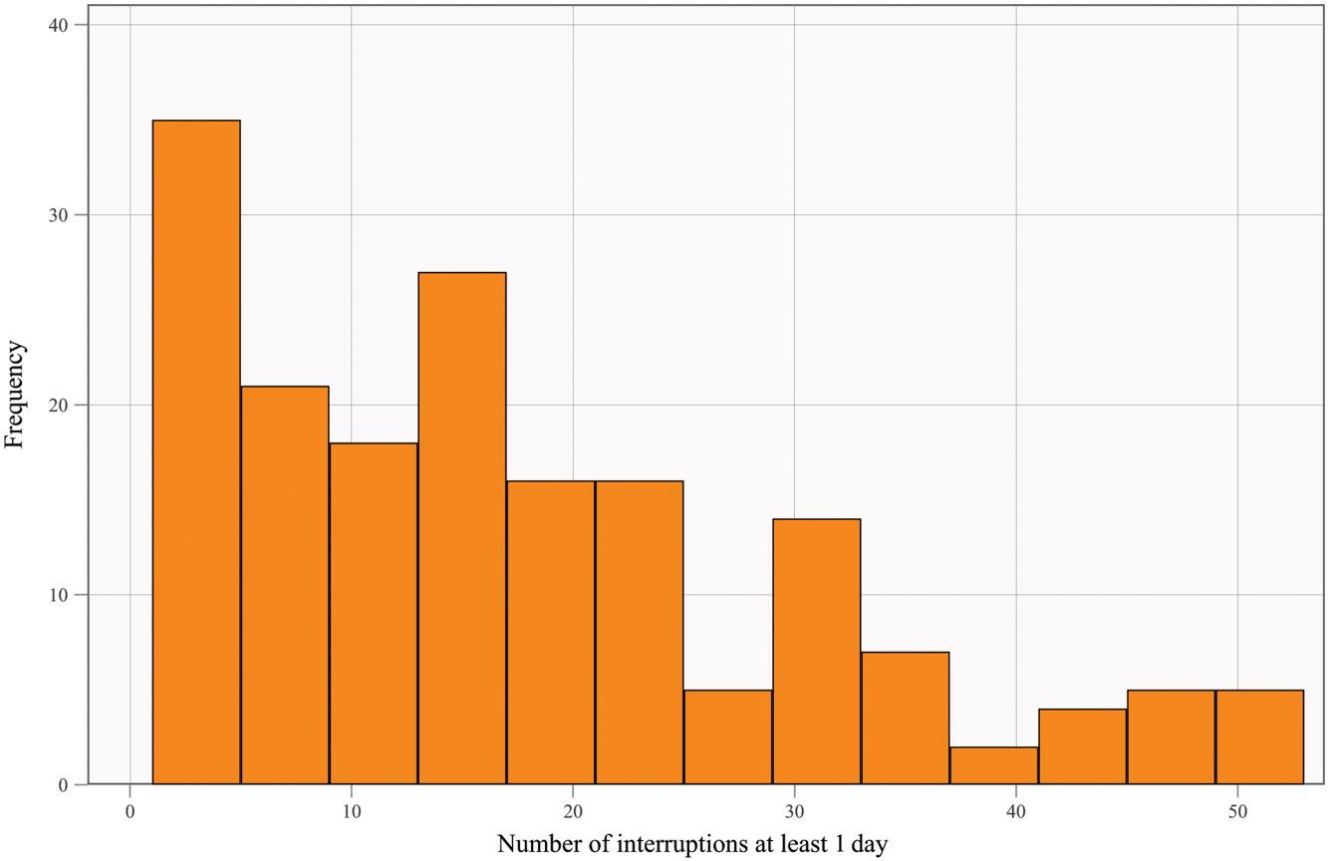
Number of treatment interruptions per participant. Of 209 participants, 175 (83.7%) had at least 1 interruption and are included in the graph while 34 (16.3%) had no interruptions and are not included in the graph.

A total of 2962 treatment interruptions were recorded among 175 of 209 (83.7%) participants, with a median of 14 (IQR, 6–24) interruptions per participant. Thirty-four of 209 (16.3%) participants had no recorded interruptions (Supplementary Table 5, Figure 4). Most interruptions (2072/2962 [70.0%]) lasted 1–2 days and 33 of 2962 (1.1%) exceeded 30 days. Treatment interruption durations were shorter in group A compared with the other 3 adherence groups, with ≤2-day interruptions occurring in 58.2% versus 4.0%–14.3% (*P* < .001 using χ^2^ test) (Supplementary Figures 5 and 6).

In adjusted multinomial logistic regression, using group A as a reference, participants receiving exclusively ambulatory care had higher odds of belonging to group B and group D. Those treated with shorter regimens only had higher odds of being in group D. Age <40 years was associated with belonging to group B and group C (Table 3).

**Table 3.** Characteristics Associated With Adherence Trajectory Groups

Participant or Treatment Characteristic	Multivariable Analysis	
	Adjusted OR (95% CI)	*P* Value
Group A	Reference	
Group B		
Ambulatory care only	13.4 (5.1–35.2)	.001
Shorter regimen only	1.8 (.6–5.4)	.28
Age <40 y	3.2 (1.3–8.2)	.013
Group C		
Ambulatory care only	1.6 (.5–4.7)	.42
Shorter regimen only	1.3 (.5–3.5)	.54
Age <40 y	2.9 (1.1–7.3)	.026
Group D		
Ambulatory care only	9.8 (4.3–22.5)	<.001
Shorter regimen only	3.0 (1.1–8.3)	.034
Age <40 y	1.2 (.5–2.6)	.68

Reference groups: Ambulatory care only: ever had hospital care; Given shorter regimen only: changed to individualized regimen; Age <40 years: age 40 years and above.

Abbreviations: CI, confidence interval; OR, odds ratio.

**Figure 5.**
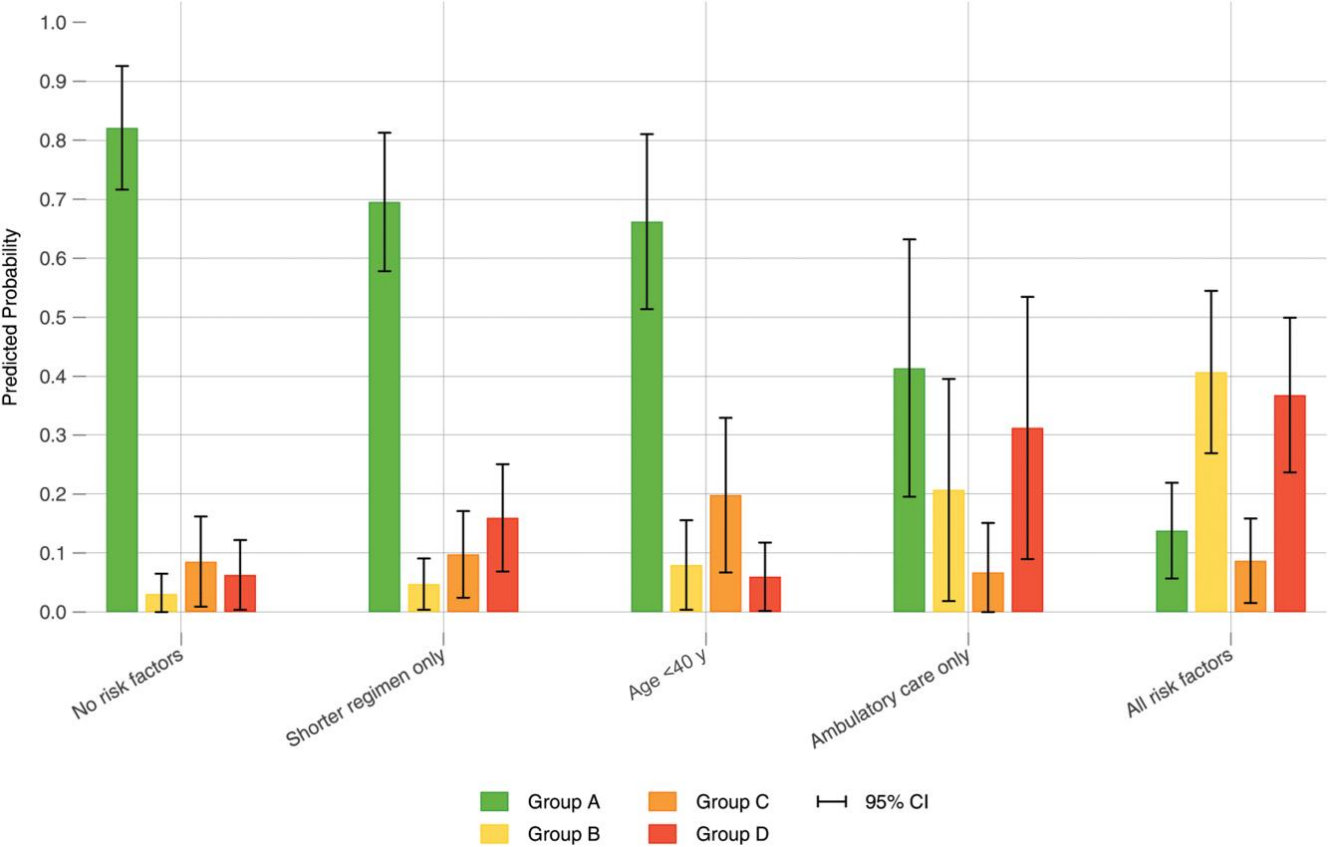
Marginal predicted probabilities of adherence trajectory group membership with patterns of characteristics. Risk factors defined as follows: shorter regimen only (given shorter regimen and never changed to an individualized regimen); age <40 years; ambulatory care only (never received in-hospital care); or all risk factors (having the combination of receipt of shorter regimen only, age <40 years, and treatment in ambulatory care only). Marginal probabilities are predicted following a multinominal logistic regression model. Negative confidence intervals (CIs) in probabilities were adjusted to zero.

Participants with a combination of ambulatory care only, age <40 years, and being treated exclusively with the shorter regimen had a 14% probability of group A membership. Probabilities for belonging to groups B and D were 41% and 37%, respectively. Individuals without any of these characteristics had an 82% probability of group A membership (Figure 5).

An analysis on the relationship between adherence and mortality was not possible as most deaths occurred early during hospital care, resulting in collinearity. There were too few participants experiencing treatment failure (14 participants [7%]) to provide a meaningful analysis for that outcome. In adjusted analysis, higher overall treatment adherence was associated with lower risk of LTFU (adjusted odds ratio [aOR], 0.85 [95% confidence interval {CI}, .75–1.00] for every 10% increase in adherence days; *P* = .048). Group D membership was strongly associated with increased risk of LTFU (aOR, 5.5 [95% CI, 2.3–13.2]; *P* < .001; Supplementary Tables 6 and 7). Higher adherence overall was associated with sustained SCC (adjusted hazard ratio, 1.19 [95% CI, 1.08–1.32]; *P* = .001 for every 10% increase in adherence days; Supplementary Table 8).

## DISCUSSION

We characterized treatment adherence to an oral RR-TB regimen in the South African TB program using DOT and digital pillboxes. Although overall adherence was >80%, treatment interruptions were frequent, and we identified 4 distinct adherence patterns where approximately 35% of participants belonged to groups with markedly decreasing adherence over time. Those with the most rapidly declining adherence trajectory had a substantially higher risk of being lost to follow-up. Participants receiving a period of inpatient treatment and an individualized regimen were more likely to belong in groups with better adherence.

Our observation of decreasing overall adherence after the first months of treatment is similar to observations in HIV and drug-susceptible TB [21, 22] and consistent with previous reports in RR-TB [7]. Similar to our cohort, these RR-TB studies used digital pillboxes or video-observed treatment to assess adherence, but did so over only 6 months and for mainly injectable-based, longer RR-TB regimens. Adherence thresholds for effective treatment in RR-TB are unknown, and clinical trials typically consider 80%–90% adherence adequate in drug-susceptible TB [23, 24]. However, this summary estimate fails to capture the complexity of temporal adherence patterns. Our latent class analysis enabled separation of adherence trajectories over time, revealing specific groups of people with more substantial declines. This is concerning, given our finding that adherence was associated with SCC and that regimen effectiveness is likely severely compromised when only 50% of doses are taken, as observed in these groups.

Treatment interruptions were highly variable, though most were brief, predominantly 1–2 days. Similar observations were reported in the Philippines [4] (median, 1.4 days) and Eastern Europe (median, 3 days) [6]. In our study, only 1% of interruptions exceeded 30 days, though these pose significant concern, as prolonged interruptions may lead to poor treatment outcomes and acquired drug resistance [4, 6]. Prolonged treatment interruptions are a particular issue for bedaquiline-based regimens because of its long elimination half-life leading to prolonged subtherapeutic exposures after discontinuation, promoting resistance development.

Using reliable and reproducible tools in adherence measurement is important for comparability and unbiased results. Limitations of self-report and pill count have been documented in previous HIV and TB studies reporting poor association with HIV virological failure [25], drug concentrations [26], and digital pillbox measures [27]. A recent study analyzing adherence in RR-TB (N = 1787) showed 4 distinct adherence trajectories with the 2 lower adherence trajectories associated with unsuccessful outcomes, although the study was limited by using self-report and pill count [9]. A strength of our study was use of digital pillboxes for adherence measurement. This tool is highly accurate and is associated with virological failure in HIV treatment [25], but has infrequently been used in RR-TB research [5, 7]. In drug-susceptible TB, lower adherence in early treatment measured by a digital pillbox has been associated with disengagement from care [21]. Use of a digital pillbox as an adherence measure for shorter 9- to 12-month RR-TB regimens is only described in a previous study [5] conducted in South Africa. In contrast to our study, adherence was measured for bedaquiline only over 6 months with analysis using a prespecified 85% cutoff, precluding detailed temporal analysis.

Risk factors for decreased adherence in previous TB studies have included male sex [20], younger age [19], higher education [20], comorbidities [18], previous TB treatment [8], receiving social grants [5], or experiencing financial hardships [18]. However, these have been inconsistent, likely reflecting differences in structural (social and healthcare related), treatment, and patient factors across settings [28]. The association between exclusive use of the shorter regimen and the worst adherence group in our study (group D) was unexpected, as we assumed that shorter treatment would enhance adherence. One potential explanation for this finding is increased pill burden in the standardized shorter regimen versus individualized longer regimens [29, 30]. Another possibility is the shorter regimen being a proxy for less severe disease since 48% had a positive sputum microscopy at baseline compared to 78% in those receiving individualized regimens. Individualized longer regimens were recommended for people with extensive chest X-ray involvement or severe extrapulmonary disease, and qualitative studies from drug-susceptible TB have shown that higher adherence motivation was seen in those experiencing more severe symptoms [28]. Differential adherence trajectories with shorter regimens may also be explained by improved understanding of the importance of treatment completion for individualized regimens, facilitating higher adherence as seen in qualitative studies [28]. We were unable to explore patients’ attitudes and beliefs influencing adherence as no qualitative data were collected.

Care setting has not previously been analyzed as an adherence predictor in RR-TB [4–7, 10]. Interpretation is challenging due to confounding, as individuals requiring hospitalization may have other characteristics affecting adherence, for example requiring more social or adherence support, or having more severe illness [29]. In our study, a period of hospitalization was associated with improved overall adherence. This could be explained by receipt of DOT while in hospital, with declining average adherence after discharge without DOT. Outpatient TB treatment offers limited opportunities for enhanced adherence and social support, possibly resulting in lower TB knowledge and trust in healthcare services [28, 29]. Hospitalized participants had lower baseline hemoglobin and albumin, suggesting increased disease severity, but these factors did not predict adherence group membership in multivariable analysis. Qualitative research is needed to understand reasons for differing adherence patterns across care settings.

Age <40 years was associated with lower overall adherence and declining adherence over time. This is consistent with a previous South African study where younger age was associated with missed RR-TB appointments [19]; however, this has not been observed in other settings [5, 7]. Younger participants may face competing responsibilities (employment and education) or less autonomy for decision-making affecting treatment prioritization, as clinic attendance involves opportunity costs (lost time or income) [5, 28, 30]. A previous study from South Africa found that receipt of a social grant was associated with <85% adherence among people with HIV and RR-TB [5]. In our study, neither socioeconomic factors nor HIV status influenced adherence trajectories, although social deprivation and HIV coinfection was universally high.

Our study had several limitations. First, our results of risk factors associated with adherence may both be underestimated (due to younger age) or overestimated (fewer received exclusive ambulatory care) since the 39 of 248 participants (16%) with unassessable adherence data were mostly lost to follow-up (which was associated with lower adherence). Second, a drawback of the digital pillbox was the need for manual data download to acquire adherence data. Illustrating this, 61.5% (24/39; Table 1) of individuals without adherence data were lost to follow-up. We were able to mitigate data loss through frequent contact attempts and home visits, recovering adherence data for two-thirds (50/74 [67.5%]) of those who were initially lost to follow-up. Technical pillbox issues, mainly limited battery life, also reduced the amount of assessable data. Third, digital pillboxes are only a surrogate for drug intake, although high accuracy has previously been shown for adherence measurement [25]. A fourth limitation was relatively small numbers in certain trajectory groups, resulting in limited precision in multinomial logistic regression modeling. Fifth, we were unable to explore the relationship between adherence and mortality as most deaths occurred early in treatment when participants were in hospital, resulting in collinearity. Finally, there were unmeasured confounders related to patients’ attitudes and beliefs, and structural and healthcare-related adherence factors; these may be better addressed through qualitative studies.

In conclusion, our study revealed 4 distinct adherence trajectories among people treated with a shorter oral regimen for RR-TB with lower adherence associated with LTFU. Risk factors associated with rapidly declining adherence may be used to identify patients requiring more intensive adherence support in TB programs. Our findings are broadly applicable within routine care using an oral RR-TB regimen as the shorter regimen is still used globally and recommended by WHO. It is unknown whether 6-month bedaquiline, pretomanid, and linezolid–based therapy, now standard of care in South Africa, will influence adherence patterns. Qualitative research exploring underlying reasons for adherence patterns is needed to further refine understanding and to identify interventions for supporting RR-TB treatment.

## Supplementary Material

ciag069_Supplementary_Data

## Supplementary Data

Supplementary materials are available at *Clinical Infectious Diseases* online. Consisting of data provided by the authors to benefit the reader, the posted materials are not copyedited and are the sole responsibility of the authors, so questions or comments should be addressed to the corresponding author.
