# Supplementary material for: Treatment Adherence With an Oral 9-Month Regimen for Rifampicin-Resistant Tuberculosis in South Africa
Source: Clin Infect Dis. 2026 Feb 9;83(1):e145–54. doi: 10.1093/cid/ciag069 (PMC13393111; doi:10.1093/cid/ciag069)
Supplement: ciag069_Supplementary_Data [file ciag069_supplementary_data.docx]

**Treatment adherence with an oral nine-month regimen for rifampicin-resistant tuberculosis in South Africa**

**SUPPLEMENTARY MATERIAL**

CONTENTS

[Supplementary methods 2](#_Toc219715065)

[Table S1: Assessable adherence days 3](#_Toc219715066)

[Table S2: Percentage of adherence overall and monthly 4](#_Toc219715067)

[Table S3: Observed adherence trajectory group membership and average posterior probabilities 5](#_Toc219715068)

[Table S4. Participant characteristics and treatment outcomes by adherence group 6](#_Toc219715069)

[Table S5: Description of treatment interruption patterns 8](#_Toc219715070)

[Table S6: Relationship between overall adherence and lost to follow-up using a logistic regression model 10](#_Toc219715071)

[Table S7: Relationship between adherence trajectory group membership and lost to follow-up using a logistic regression model 11](#_Toc219715072)

[Table S8: Relationship between overall adherence and sustained sputum culture conversion using a Cox regression model 12](#_Toc219715073)

[Figure S1: Included participants in the SHIFT-TB cohort with assessable adherence data 13](#_Toc219715074)

[Figure S2: Individual observed monthly adherence over nine months by adherence trajectory group 14](#_Toc219715075)

[Figure S3: Adherence trajectories over nine months - sensitivity analysis including data from the digital pillbox only 15](#_Toc219715076)

[Figure S4: Adherence trajectories over nine months - sensitivity analysis including those with ≥75% assessable adherence data only 16](#_Toc219715077)

[Figure S5: Treatment interruption patterns by adherence trajectory group in participants with at least one interruption 17](#_Toc219715078)

[Figure S6: Gaps between treatment interruptions by adherence trajectory group in participants with at least one interruption 18](#_Toc219715079)

[References 19](#_Toc219715080)

## Supplementary methods

Modified World Health Organization [1] outcomes included standard definitions except that treatment failure due to lack of sputum culture conversion (SCC) or reversion was assigned without regimen change and that lack of SCC was assessed at six months.

Time to sustained sputum culture conversion (SCC) was defined as time from treatment initiation to the first of two negative sputum cultures, consecutive or not but without a positive sputum in between and not followed by a positive sputum culture until end of follow-up.

Adherence in participants lost to follow-up was measured until end of drug treatment, not including the last two months used to define the treatment outcome.

The latent class analysis employed group-based trajectory modelling using the Stata traj plug-in [2], a finite mixture model application assigning trajectory group membership based on highest posterior probabilities. Expected group proportions are also estimated. A maximum of four groups was assumed to capture clinically relevant adherence scenarios. The individual and summary median observed monthly adherence data were viewed to decide on potential model parameters (e.g., linear or quadratic). Modelling approach, number of trajectory groups and model fit were based on criteria as outlined by Nguena et al [3]. Model development began with a one-group scenario including a quadratic parameter. If the model parameter was statistically significant, groups were added progressively until one additional group (five groups) than the clinically relevant scenario. If the quadratic parameter was nonsignificant, a linear parameter followed by a cubic parameter were tested. Model fit was evaluated using Bayesian Information Criterion (BIC) [4], average posterior probabilities ≥0.7 per group, entropy ≥0.8 (model ability to distinguish separate groups), and ≥5% participants per group [3].

Bayesian Information Criterion (BIC) was used when evaluating model fit in the group-based trajectory model. The BIC difference between two models times 2 was used as an estimate of the log of the Bayes factor [5]. A calculated difference of 2 or more were considered evidence of a model difference.

## Table S1: Assessable adherence days

| Adherence availability | Digital pillbox and DOT,  median % (IQR) | Digital pillbox,  median % (IQR) |
| --- | --- | --- |
| Assessable adherence days overall | 95 (76-100), n=209 | 92 (67-100), n=184 |
| Assessable adherence days per month |  |  |
| Month 1 | 100 (80-100), n=209 | 57^a^ (47-100), n=109 |
| Month 2 | 100 (100-100), n=204 | 100 (100-100), n=131 |
| Month 3 | 100 (100-100), n=189 | 100 (97-100), n=156 |
| Month 4 | 100 (100-100), n=177 | 100 (100-100), n=167 |
| Month 5 | 100 (100-100), n=167 | 100 (100-100), n=164 |
| Month 6 | 100 (100-100), n=157 | 100 (100-100), n=154 |
| Month 7 | 100 (73-100), n=151 | 100 (73-100), n=149 |
| Month 8 | 100 (66-100), n=141 | 100 (53-100), n=139 |
| Month 9 | 100 (37-100), n=138 | 100 (37-100), n=137 |

DOT, directly observed treatment; IQR = Interquartile range. One month = 30 days.
An assessable adherence day was defined as a day with a daily electronic signal (recorded by the digital pillbox to ensure function) when drugs were prescribed. Proportion assessable adherence days were reported as all assessable adherence days over total adherence days when drugs were prescribed. a The digital pillbox was given to participants up to 30 days after treatment initiation.

## Table S2: Percentage of adherence overall and monthly

| Adherence | Digital pillbox and DOT,  median % (IQR) | Digital pillbox,  median % (IQR) |
| --- | --- | --- |
| Adherence overall | 82 (63-98), n=209 | 72 (51-92), n=184 |
| Adherence monthly |  |  |
| Month 1 | 100 (94-100), n=207 | 94 (82-100), n=95 |
| Month 2 | 100 (86-100), n=201 | 90 (63-100), n=124 |
| Month 3 | 97 (67-100), n=181 | 90 (57-100), n=143 |
| Month 4 | 87 (57-100), n=156 | 83 (48-100), n=144 |
| Month 5 | 83 (43-97), n=149 | 77 (40-97), n=145 |
| Month 6 | 73 (33-97), n=137 | 73 (33-93), n=133 |
| Month 7 | 63 (30-92), n=135 | 63 (30-90), n=133 |
| Month 8 | 60 (30-90), n=113 | 58 (30-87), n=110 |
| Month 9 | 55 (21-83), n=105 | 53 (18-83), n=104 |

DOT, directly observed treatment; IQR = Interquartile range. One month = 30 days. Adherence is calculated over all assessable adherence days (e.g., days with an electronic signal confirming function from the digital pillbox or directly observed treatment days).

## Table S3: Observed adherence trajectory group membership and average posterior probabilities

| Trajectory group | Participants included in each trajectory group, n=209,  n (%) | Average posterior probabilities |
| --- | --- | --- |
| **Group A** | 101 (48.3) | 0.86 |
| **Group B** | 35 (16.8) | 0.86 |
| **Group C** | 25 (12.0) | 0.90 |
| **Group D** | 48 (23.0) | 0.87 |

## Table S4. Participant characteristics and treatment outcomes by adherence group

| Characteristic | Overall  N = 209^a^,  n (%) | Group A  n=101,  n (%) | Group B  n=35,  n (%) | Group C  n=25,  n (%) | Group D  n=48,  n (%) |
| --- | --- | --- | --- | --- | --- |
| Female sex | 81 (38.8) | 38 (37.6) | 13 (37.1) | 11 (44.0) | 19 (39.6) |
| Below 40 years | 108 (51.7) | 43 (42.6) | 25 (71.4) | 17 (68.0) | 23 (47.9) |
| HIV test positive | 140 (67.0) | 69 (68.3) | 25 (71.4) | 18 (72.0) | 28 (58.3) |
| Previous TB disease | 99 (47.4) | 47 (46.5) | 19 (54.3) | 15 (57.7) | 18 (38.3) |
| Positive microscopy | 117 (56.0), n=207 | 62 (61.4), n=101 | 17 (48.6), n=35 | 17 (68.0), n=25 | 21 (43.8), n=46 |
| Positive sputum culture | 168 (80.4), n=206 | 83 (82.2), n=100 | 29 (82.9), n=35 | 19 (76.0), n=25 | 37 (77.1), n=46 |
| Baseline haemoglobin (g/dL), mean (95% CI) | 10.8 (10.5-11.1), n=207 | 10.2 (9.8-10.7), n=100 | 11.2 (10.4-11.9), n=35 | 10.8 (10.0-11.7), n=25 | 11.5 (10.8-12.3), n=47 |
| Baseline albumin (g/L), mean (95% CI) | 30 (29-31), n=201 | 28 (26-29), n=99 | 33 (30-35), n=32 | 31 (28-33), n=25 | 33 (31-35), n=45 |
| Single | 176 (84.2) | 80 (79.2) | 31 (88.6) | 24 (96.0) | 41 (85.4) |
| Working or studying | 75 (35.9) | 34 (33.7) | 14 (40.0) | 7 (28.0) | 20 (41.7) |
| Education level |  |  |  |  |  |
| No education/ primary only | 51 (24.4) | 27 (26.7) | 8 (22.9) | 5 (20.0) | 11 (22.9) |
| High school/University | 158 (75.6) | 74 (73.3) | 27 (77.1) | 20 (80.0) | 37 (77.1) |
| Received a social grant | 75 (35.9) | 35 (34.7) | 8 (22.9) | 11 (44.0) | 21 (43.8) |
| CAGE score ≥ 2 |  |  |  |  |  |
| No | 66 (31.6) | 33 (32.7) | 10 (28.6) | 8 (32.0) | 15 (31.3) |
| Yes | 107 (51.2) | 48 (47.5) | 20 (57.1) | 11 (44.0) | 28 (58.3) |
| Missing | 36 (17.2) | 20 (19.8) | 5 (14.3) | 6 (24.0) | 5 (10.4) |
| Ambulatory care only | 81 (38.8) | 16 (15.8) | 26 (74.3) | 6 (24.0) | 33 (68.8) |
| Shorter regimen only | 149 (71.3) | 61 (60.4) | 29 (82.9) | 17 (68.0) | 42 (87.5) |
| Treatment length total regimen (days), median (IQR) | 292 (182-353) | 292 (120-412) | 295 (261-331) | 303 (283-353) | 283 (154-331) |
| Sustained SCC | 156 (92.9), n=168 | 76 (91.6), n=83 | 29 (100), n=29 | 18 (94.7), n=19 | 33 (89.2), n=37 |
| Time to sustained SCC (days), median (IQR) | 29 (16-42), n=156 | 27 (18-42), n=76 | 29 (15-46), n=29 | 29 (21-42), n=18 | 29 (14-42), n=33 |
| Treatment outcome |  |  |  |  |  |
| Treatment success | 118 (56.5) | 54 (53.5) | 23 (65.7) | 18 (72.0) | 23 (47.9) |
| Treatment failed | 14 (6.7) | 7 (6.9) | 4 (11.4) | 0 (0) | 3 (6.3) |
| Lost to follow-up | 50 (23.9) | 16 (15.8) | 8 (22.9) | 7 (26.9) | 19 (40.4) |
| Died | 27 (12.9) | 24 (23.8) | 0 (0) | 1 (4.0) | 2 (4.2) |

CI, confidence interval; IQR, interquartile range; SCC, sputum culture conversion. CAGE is a four-question screening tool for alcohol assessment. a participants with assessable adherence data.

## Table S5: Description of treatment interruption patterns

| Treatment interruption patterns and gaps between interruptions | n (%) |
| --- | --- |
| Interruption frequency |  |
| Total frequency | 2962 (100) |
| Median (IQR, range) per patient | 14 (6-24, 1-53) |
| Interruption frequency per treatment time period |  |
| 1-3 months | 792 (26.7) |
| 4-6 months | 1100 (37.1) |
| 7-9 months | 1070 (36.1) |
| Interruption duration |  |
| Median (IQR, range) total (days) | 1 (1-3, 1-156) |
| Median (IQR, range) per patient (days) | 1 (1-2, 1-46) |
| Maximum (IQR, range) per patient (days) | 8 (2-19, 1-156) |
| Total (IQR, range) per patient (days) | 30 (3-75, 0-193) |
| Interruption duration by categories |  |
| 1-2 days | 2072 (70.0) |
| 3-7 days | 650 (21.9) |
| 8-14 days | 134 (4.5) |
| 15-30 days | 73 (2.5) |
| >30 days^a^ | 33 (1.1) |
| Maximum interruption duration per patient by categories |  |
| 1-2 days | 48 (27.4) |
| 3-7 days | 39 (22.3) |
| 8-14 days | 30 (17.1) |
| 15-30 days | 20 (17.1) |
| >30 days | 28 (16.0) |
| Time to interruption |  |
| Median (IQR, range) days | 147 (85-204, 1-270) |
| Median (IQR, range) days per patient | 145 (87-186, 1-270) |
| Median (IQR, range) shortest days per patient | 52 (17-111, 1-270) |
| Medium (IQR, range) interruption duration per treatment time period (days) |  |
| 1-3 months | 1 (1-2, 1-50) |
| 4-6 months | 1 (1-3, 1-156) |
| 7-9 months | 2 (1-3, 1-72) |
| Duration gap between interruptions |  |
| Medium (IQR, range) days | 3 (1-7, 1-257) |
| Medium (IQR, range) days per patient | 4 (2-10, 1-134) |
| Maximum (IQR, range) days per patient | 55 (20-101, 1-257) |
| Medium duration gap between interruptions per patient |  |
| <10 days | 132 (75.4) |
| 10 days or more | 43 (24.6) |

IQR, interquartile range. 175/209 (83.7%) participants had at least one interruption, while 34/209 (16.3%) had no interruptions. a 28 patients with one interruption and 5 patients with two interruptions.

## Table S6: Relationship between overall adherence and lost to follow-up using a logistic regression model

| Participant or treatment characteristics |  |  | Univariable analysis | | Multivariable analysis | |
| --- | --- | --- | --- | --- | --- | --- |
|  | **Not LTFU (n=159),**  **n (%)** | **LTFU  (n=50),**  **n (%)** | **Crude OR (95% CI)** | **p-value** | **Adjusted OR (95% CI)** | **p-value** |
| Overall adherence, median (IQR) | 84.7  (63.6-98.4) | 74.1  (53.5-94.8) | 0.87 (0.76-1.00)^a^ | 0.052 | 0.86 (0.75-1.00)^a^ | 0.048 |
| Female sex | 66 (41.5) | 15 (30.0) | 0.60 (0.31-1.19) | 0.15 |  |  |
| Below 40 years | 75 (47.2) | 33 (66.0) | 2.17 (1.12-4.22) | 0.022 |  |  |
| HIV test positive | 108 (67.9) | 32 (64.0) | 0.84 (0.43-1.64) | 0.61 |  |  |
| Previous TB disease | 75 (47.2) | 24 (48.0) | 1.03 (0.55-1.95) | 0.92 |  |  |
| Positive microscopy, n=207 | 87 (54.7) | 30 (60.0) | 1.29 (0.67-2.48) | 0.45 |  |  |
| Baseline haemoglobin (g/dL), mean (95% CI), n=207 | 10.7 (10.3-11.1) | 11.0 (10.3-11.7) | 1.05 (0.92-1.21) | 0.47 |  |  |
| Baseline albumin (g/L), mean (95% CI), n=201 | 30 (29-31) | 31 (29-33) | 1.02 (0.97-1.07) | 0.40 |  |  |
| Single | 128 (80.5) | 48 (96.0) | 5.81 (1.34-25.2) | 0.019 | 6.37 (1.43-28.4) | 0.015 |
| Working/studying | 57 (35.8) | 18 (36.0) | 1.01 (0.52-1.95) | 0.99 |  |  |
| Education level |  |  |  |  |  |  |
| No education/ primary care | 40 (25.2) | 11 (22.0) | 1.00 | - |  |  |
| High school/University | 119 (74.8) | 39 (78.0) | 1.19 (0.56-2.55) | 0.65 |  |  |
| Received a social grant | 56 (35.2) | 19 (38.0) | 1.13 (0.58-2.18) | 0.72 |  |  |
| CAGE score ≥2 |  |  |  |  |  |  |
| No | 49 (30.8) | 17 (34.0) | 1.00 | - |  |  |
| Yes | 80 (50.3) | 27 (54.0) | 0.97 (0.48-1.97) | 0.94 |  |  |
| Missing | 30 (18.9) | 6 (12.0) | 0.58 (0.20-1.62) | 0.30 |  |  |
| Ambulatory care only | 57 (35.8) | 24 (48.0) | 1.65 (0.87-3.14) | 0.13 |  |  |
| Shorter regimen only | 118 (74.2) | 31 (62.0) | 0.57 (0.29-1.11) | 0.098 | 0.40 (0.20-0.83) | 0.014 |

CI, confidence interval; IQR, interquartile range; LTFU, lost to follow-up; OR, odds ratio. a in 10 percentage points steps.

## Table S7: Relationship between adherence trajectory group membership and lost to follow-up using a logistic regression model

| Participant or treatment characteristics |  |  | Univariable analysis | | Multivariable analysis | |
| --- | --- | --- | --- | --- | --- | --- |
|  | **Not LTFU (n=159), n (%)** | **LTFU (n=50), n (%)** | **Crude OR (95% CI)** | **p-value** | **Adjusted OR (95% CI)** | **p-value** |
| Trajectory group |  |  |  |  |  |  |
| Group A | 85 (53.5) | 16 (32.0) | 1.00 | - | 1.00 | - |
| Group B | 27 (17.0) | 8 (16.0) | 1.57 (0.61-4.08) | 0.35 | 1.84 (0.67-5.07) | 0.24 |
| Group C | 19 (11.9) | 6 (12.0) | 1.68 (0.58-4.85) | 0.34 | 1.53 (0.51-4.64) | 0.45 |
| Group D | 28 (17.6) | 20 (40.0) | 3.79 (1.73-8.31) | 0.001 | 5.47 (2.26-13.2) | <0.001 |
| Female sex | 66 (41.5) | 15 (30.0) | 0.60 (0.31-1.19) | 0.15 |  |  |
| Below 40 years | 75 (47.2) | 33 (66.0) | 2.17 (1.12-4.22) | 0.022 |  |  |
| HIV test positive | 108 (67.9) | 32 (64.0) | 0.84 (0.43-1.64) | 0.61 |  |  |
| Previous TB disease | 75 (47.2) | 24 (48.0) | 1.03 (0.55-1.95) | 0.92 |  |  |
| Positive microscopy, n=207 | 87 (54.7) | 30 (60.0) | 1.29 (0.67-2.48) | 0.45 |  |  |
| Baseline haemoglobin (g/dL), mean (95% CI), n=207 | 10.7 (10.3-11.1) | 11.0 (10.3-11.7) | 1.05 (0.92-1.20) | 0.47 |  |  |
| Baseline albumin (g/L), mean (95% CI), n=201 | 30 (29-31) | 31 (29-33) | 1.02 (0.97-1.07) | 0.40 |  |  |
| Single | 128 (80.5) | 48 (96.0) | 5.81 (1.34-25.2) | 0.019 | 7.22 (1.59-32.9) | 0.011 |
| Working/studying | 57 (35.8) | 18 (36.0) | 1.01 (0.52-1.95) | 0.99 |  |  |
| Education level |  |  |  |  |  |  |
| No education/  primary school | 40 (25.2) | 11 (22.0) | 1.00 | - |  |  |
| High school/ University | 119 (74.8) | 39 (78.0) | 1.19 (0.56-2.55) | 0.65 |  |  |
| Received a social grant | 56 (35.2) | 19 (38.0) | 1.13 (0.58-2.18) | 0.72 |  |  |
| CAGE score ≥2 |  |  |  |  |  |  |
| No | 49 (30.8) | 17 (34.0) | 1.00 | - |  |  |
| Yes | 80 (50.3) | 27 (54.0) | 0.97 (0.48-1.97) | 0.939 |  |  |
| Missing | 30 (18.9) | 6 (12.0) | 0.58 (0.20-1.62) | 0.297 |  |  |
| Ambulatory care only | 57 (35.8) | 24 (48.0) | 1.65 (0.87-3.14) | 0.13 |  |  |
| Shorter regimen only | 118 (74.2) | 31 (62.0) | 0.57 (0.29-1.11) | 0.098 | 0.30 (0.14-0.66) | 0.003 |

CI, confidence interval; IQR, interquartile range; LTFU, lost to follow-up; OR, odds ratio.

## Table S8: Relationship between overall adherence and sustained sputum culture conversion using a Cox regression model

| Participant or treatment characteristics |  |  | Univariable analysis | | Multivariable analysis | |
| --- | --- | --- | --- | --- | --- | --- |
|  | **SCC Events**  **(n=156)** | **Total**  **(n=168)** | **Crude HR (95% CI)** | **p-value** | **Adjusted HR (95% CI)** | **p-value** |
| Overall adherence | 154 | 166 | 1.18^a^ (1.06-1.30) | 0.002 | 1.19 (1.08-1.32)^a^ | 0.001 |
| Female sex | 156 | 168 | 1.18 (0.86-1.63) | 0.31 |  |  |
| Below 40 years | 156 | 168 | 1.32 (0.96-1.81) | 0.087 |  |  |
| HIV test positive |  | 166 | 1.08 (0.78-1.50) | 0.637 |  |  |
| Previous TB disease | 156 | 168 | 1.05 (0.76-1.44) | 0.78 |  |  |
| Positive microscopy | 156 | 168 | 0.79 (0.56-1.11) | 0.17 |  |  |
| Baseline haemoglobin (g/dL) | 154 | 166 | 1.01 (0.93-1.09) | 0.85 |  |  |
| Baseline albumin (g/L) | 151 | 162 | 1.00 (0.98-1.02) | 0.79 |  |  |
| Single | 156 | 168 | 1.61 (1.03-2.54) | 0.038 | 1.81 (1.14-2.87) | 0.011 |
| Working/studying | 156 | 168 | 0.99 (0.72-1.38) | 0.976 |  |  |
| Education level | 156 | 168 |  |  |  |  |
| No education/ primary school |  |  | Reference | - |  |  |
| High school/University |  |  | 1.16 (0.80-1.68) | 0.43 |  |  |
| Received a social grant | 156 | 168 | 0.78 (0.56-1.08) | 0.13 |  |  |
| CAGE score ≥2 | 156 | 168 |  |  |  |  |
| No |  |  | Reference | - | Reference | - |
| Yes |  |  | 1.29 (0.90-1.85) | 0.17 | 1.46 (1.01-2.12) | 0.044 |
| Missing |  |  | 1.56 (0.98-2.49) | 0.064 | 1.53 (0.96-2.45) | 0.077 |
| Ambulatory care only | 156 | 168 | 0.80 (0.57-1.11) | 0.18 |  |  |
| Shorter regimen only | 156 | 168 | 1.23 (0.88-1.71) | 0.23 |  |  |

CI, confidence interval; HR, hazard ratio; SCC, sputum culture conversion. Sustained SCC is defined as at least two negative sputum cultures, consecutive or not without a positive sputum culture in between and not followed by a positive sputum culture over 12 months. Overall adherence is measured until time to SCC or censoring (end of follow-up). a in 10 percentage points steps.


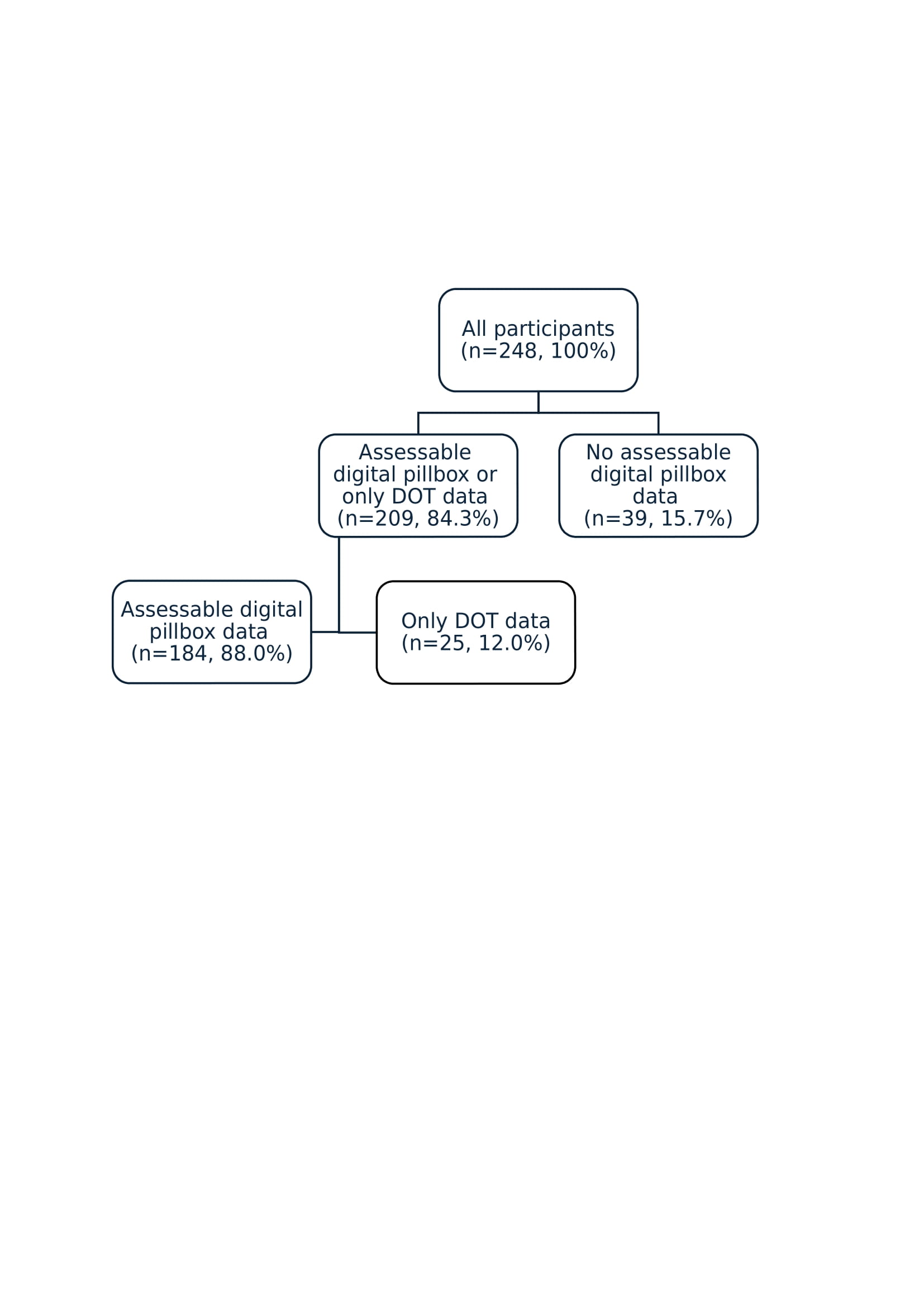


## Figure S1: Included participants in the SHIFT-TB cohort with assessable adherence data

DOT, directly observed treatment.


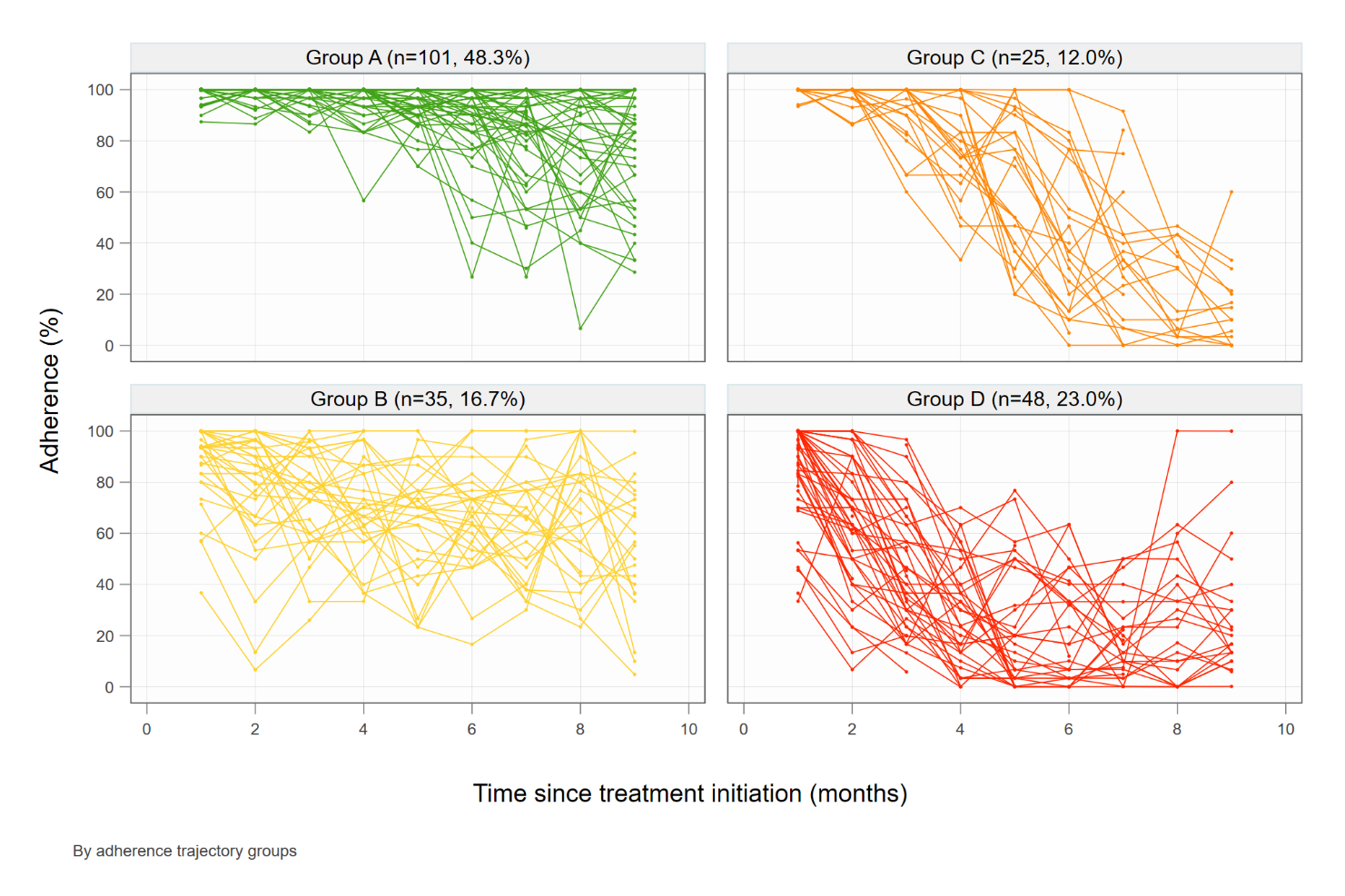


Figure S2: Individual observed monthly adherence over nine months by adherence trajectory group


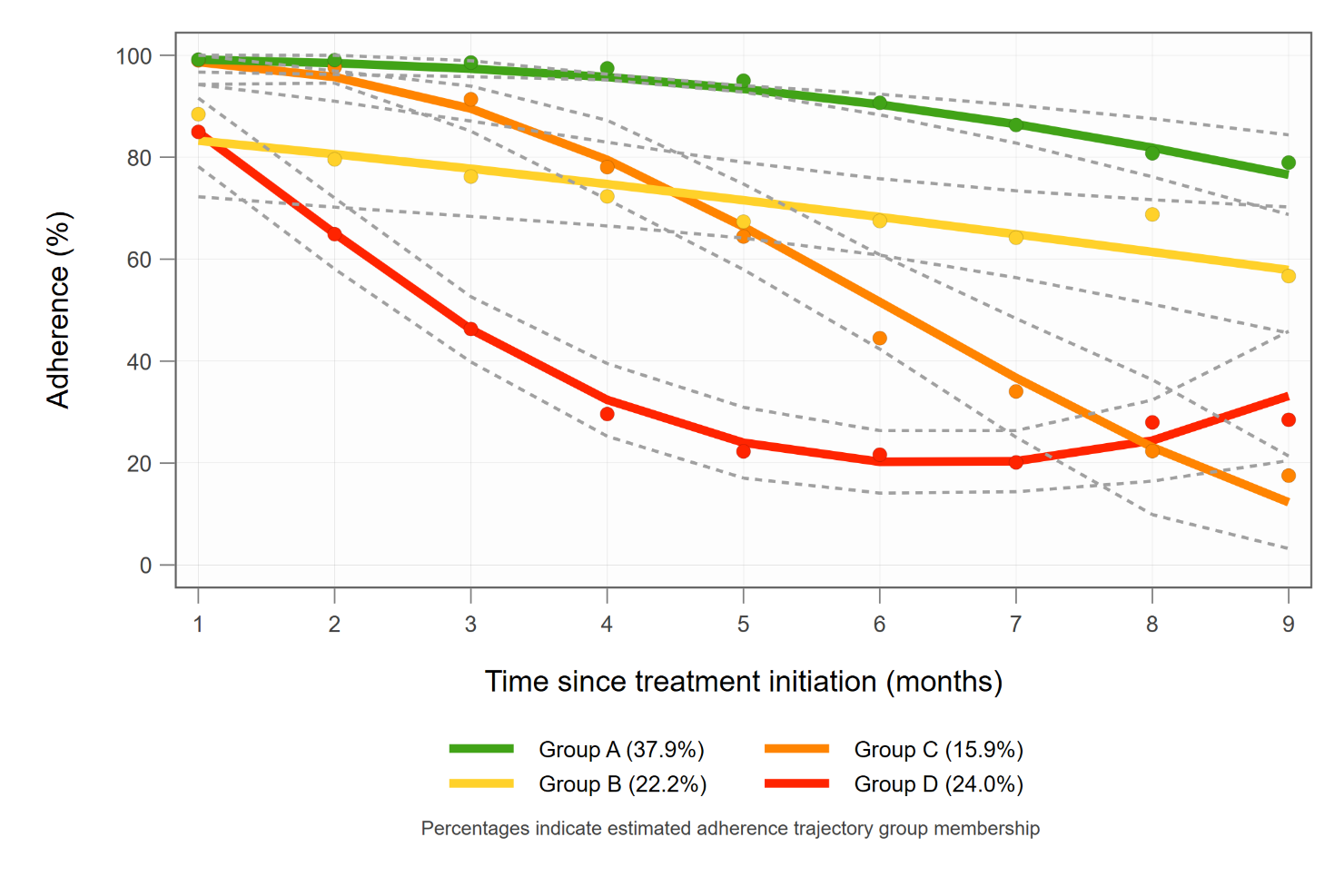


## Figure S3: Adherence trajectories over nine months - sensitivity analysis including data from the digital pillbox only

Solid lines are estimated trajectories. Dotted lines indicate 95% confidence intervals. Dots represent observed group means at different months.


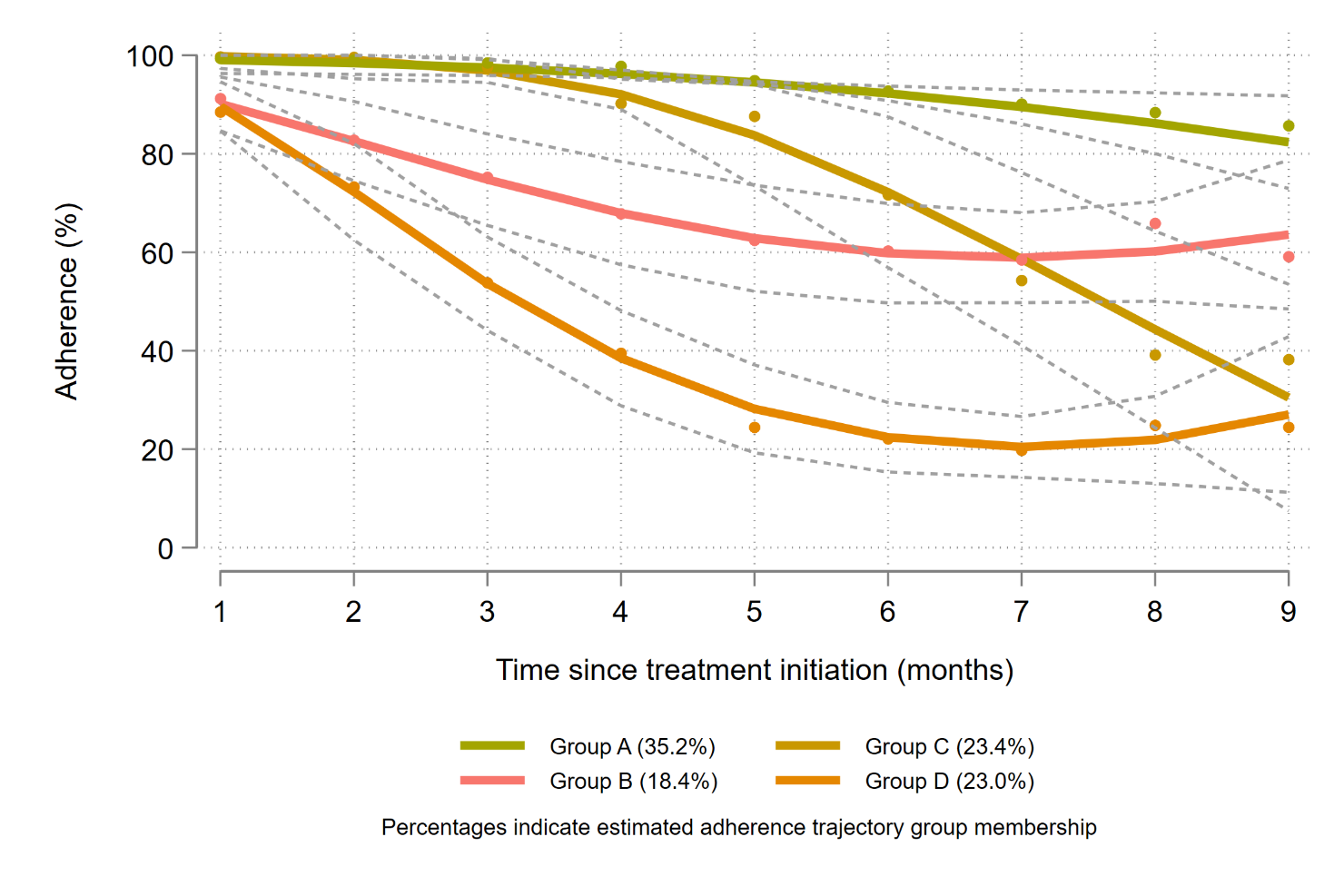


## Figure S4: Adherence trajectories over nine months - sensitivity analysis including those with ≥75% assessable adherence data only

Solid lines are estimated trajectories. Dotted lines indicate 95% confidence intervals. Dots represent observed group means at different months.


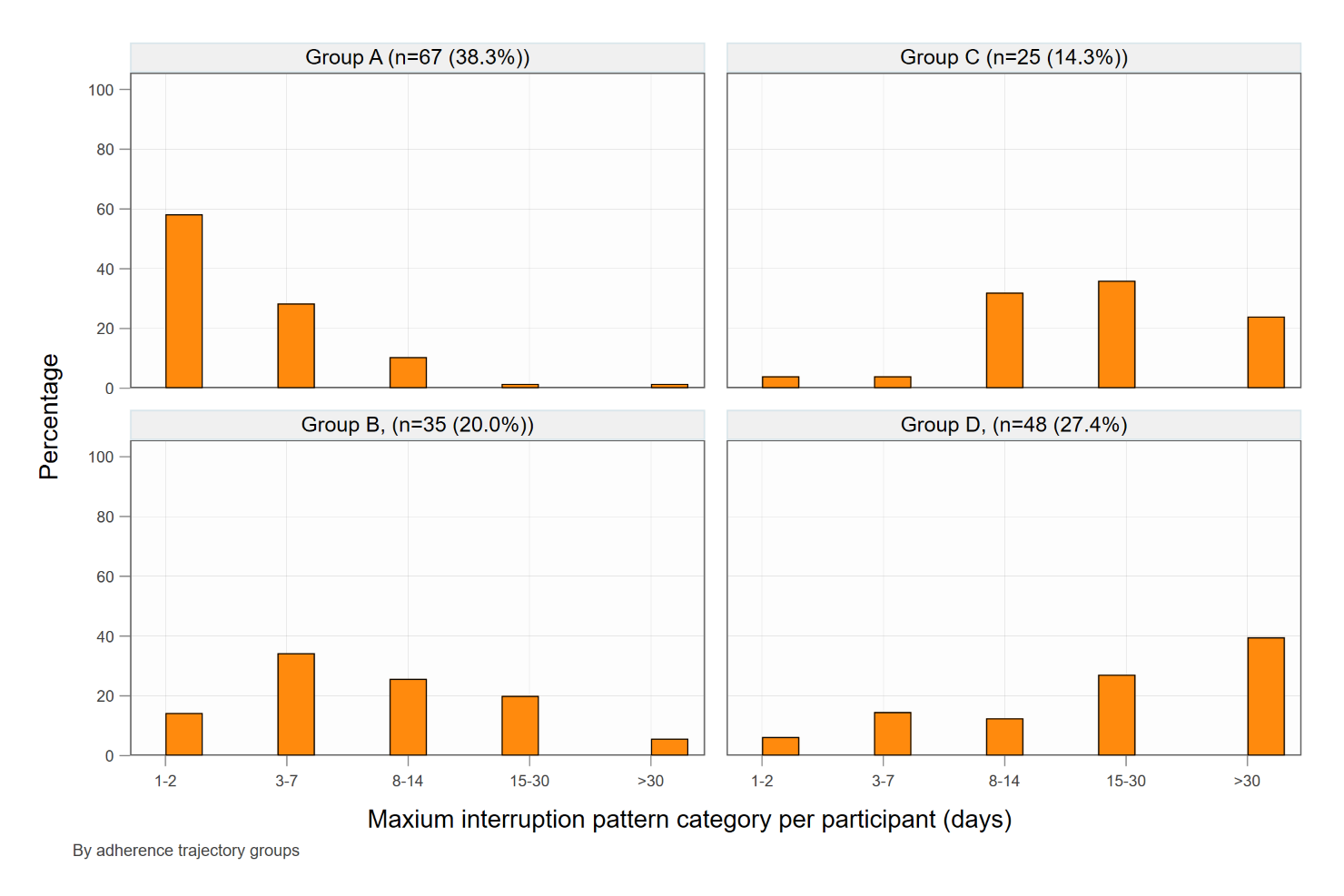


## Figure S5: Treatment interruption patterns by adherence trajectory group in participants with at least one interruption

175/209 (83.7%) participants had at least one interruption and are included in the graph while 34/209 (16.3%) had no interruptions and are not included in the graph. All 34 participants belonged to trajectory group A.


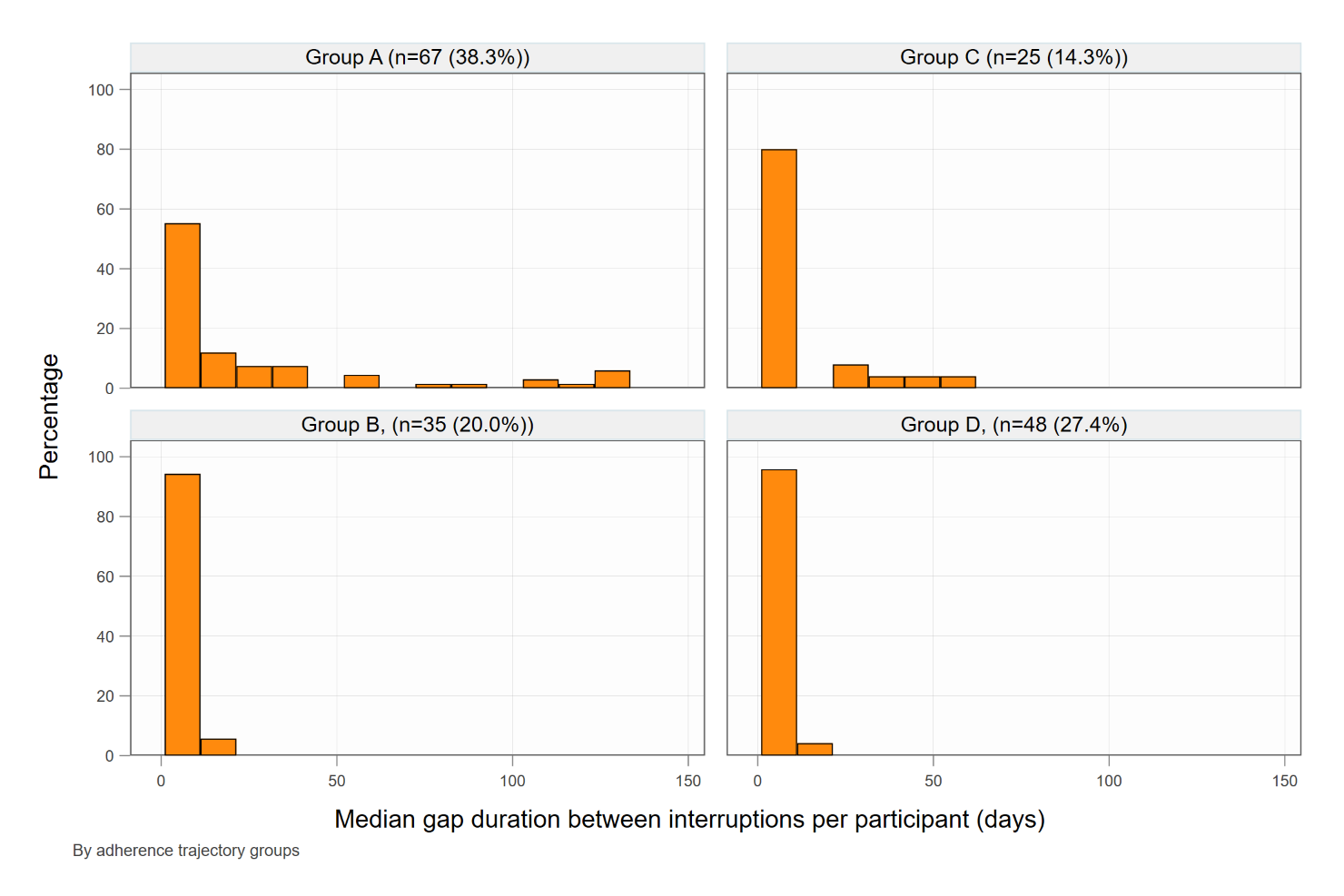


## Figure S6: Gaps between treatment interruptions by adherence trajectory group in participants with at least one interruption

175/209 (83.7%) participants had at least one interruption and are included in the graph while 34/209 (16.3%) had no interruptions and are not included in the graph. All 34 participants belonged to trajectory group A.

.

## References

1. World Health Organization. WHO operational handbook on tuberculosis. Module 4: Treatment - drug-resistant tuberculosis treatment, 2022 update. Available at: <https://www.who.int/publications/i/item/9789240065116>. Accessed 10 November 2024.

2. Jones BL, Nagin DS. A Note on a Stata Plugin for Estimating Group-based Trajectory Models. Sociological Methods & Research **2013**; 42(4): 608-13.

3. Nguena Nguefack HL, Pagé MG, Katz J, et al. Trajectory Modelling Techniques Useful to Epidemiological Research: A Comparative Narrative Review of Approaches. Clin Epidemiol **2020**; 12: 1205-22.

4. Jones BL, Nagin DS, Roeder K. A SAS Procedure Based on Mixture Models for Estimating Developmental Trajectories. Sociological Methods & Research **2001**; 29(3): 374-93.

5. Jones ASK, Bidad N, Horne R, et al. Determinants of non-adherence to anti-TB treatment in high income, low TB incidence settings: a scoping review. Int J Tuberc Lung Dis **2021**; 25(6): 483-90.
